# Supplementary material for: Biopsychosocial factors are associated with impaired sexual function in Mexican patients with rheumatoid arthritis
Source: PLoS One. 2024 Aug 26;19(8):e0305831. doi: 10.1371/journal.pone.0305831 (PMC11346937; doi:10.1371/journal.pone.0305831)
Supplement: S1 Appendix — (PDF) [file pone.0305831.s001.pdf]

Appendix: STROBE checklist for cross sectional studies for paper "Sexual and reproductive health in Mexican patients with rheumatoid arthritis during the COVID-19 pandemic: A comprehensive approach from the biopsychosocial path".

STROBE Statement—Checklist of items that should be included in reports of ***cross-sectional studies***

	Item No	Recommendation	Check
**Title and abstract**	1	(*a*) Indicate the study's design with a commonly used term in the title or the abstract	Done
		(*b*) Provide in the abstract an informative and balanced summary of what was done and what was found	Done
**Introduction**			
Background/rationale	2	Explain the scientific background and rationale for the investigation being reported	Done
Objectives	3	State specific objectives, including any prespecified hypotheses	Done
**Methods**			
Study design	4	Present key elements of study design early in the paper	Done
Setting	5	Describe the setting, locations, and relevant dates, including periods of recruitment, exposure, follow-up, and data collection	Done
Participants	6	(*a*) Give the eligibility criteria, and the sources and methods of selection of participants	Done
Variables	7	Clearly define all outcomes, exposures, predictors, potential confounders, and effect modifiers. Give diagnostic criteria, if applicable	Done
Data sources/ measurement	8*	For each variable of interest, give sources of data and details of methods of assessment (measurement). Describe comparability of assessment methods if there is more than one group	Done
Bias	9	Describe any efforts to address potential sources of bias	Partially done
Study size	10	Explain how the study size was arrived at	Done
Quantitative variables	11	Explain how quantitative variables were handled in the analyses. If applicable, describe which groupings were chosen and why	Done
Statistical methods	12	(*a*) Describe all statistical methods, including those used to control for confounding	Done
		(*b*) Describe any methods used to examine subgroups and interactions	Done
		(*c*) Explain how missing data were addressed	Done
		(*d*) If applicable, describe analytical methods taking account of sampling strategy	NA
		(*e*) Describe any sensitivity analyses	NA

**Results**			
Participants	13*	(a) Report numbers of individuals at each stage of study—eg numbers potentially eligible, examined for eligibility, confirmed eligible, included in the study, completing follow-up, and analysed	Done
		(b) Give reasons for non-participation at each stage	NA
		(c) Consider use of a flow diagram	Not done
Descriptive data	14*	(a) Give characteristics of study participants (eg demographic, clinical, social) and information on exposures and potential confounders	Done
		(b) Indicate number of participants with missing data for each variable of interest	Done
Outcome data	15*	Report numbers of outcome events or summary measures	Done
Main results	16	(*a*) Give unadjusted estimates and, if applicable, confounder-adjusted estimates and their precision (eg, 95% confidence interval). Make clear which confounders were adjusted for and why they were included	Done
		(*b*) Report category boundaries when continuous variables were categorized	Done
		(*c*) If relevant, consider translating estimates of relative risk into absolute risk for a meaningful time period	NA
Other analyses	17	Report other analyses done—eg analyses of subgroups and interactions, and sensitivity analyses	NA
**Discussion**			
Key results	18	Summarise key results with reference to study objectives	Done
Limitations	19	Discuss limitations of the study, taking into account sources of potential bias or imprecision. Discuss both direction and magnitude of any potential bias	Done
Interpretation	20	Give a cautious overall interpretation of results considering objectives, limitations, multiplicity of analyses, results from similar studies, and other relevant evidence	Done
Generalisability	21	Discuss the generalisability (external validity) of the study results	Done
**Other information**			
Funding	22	Give the source of funding and the role of the funders for the present study and, if applicable, for the original study on which the present article is based	Done

*Give information separately for exposed and unexposed groups.
