# Supplementary material for: Biopsychosocial factors are associated with impaired sexual function in Mexican patients with rheumatoid arthritis
Source: PLoS One. 2024 Aug 26;19(8):e0305831. doi: 10.1371/journal.pone.0305831 (PMC11346937; doi:10.1371/journal.pone.0305831)
Supplement: S2 Appendix — (PDF) [file pone.0305831.s002.pdf]

COVID-19 SURVEY (SPANISH VERSION)

El Departamento de Inmunología y Reumatología del Instituto Nacional de Ciencias Médicas y de la Nutrición Salvador Zubirán, está interesado en conocer algunos aspectos sobre su estado de salud en general y de la enfermedad reumatológica que usted padece, que se pudieron haber dado durante la pandemia de COVID-19. También queremos conocer, cómo ha resuelto usted los problemas de salud que pudiera haber presentado durante el periodo en el cual, el Instituto se transformó en un hospital para atender sólo COVID-19.
Por favor, complete los datos siguientes:

Fecha: ______________________

A continuación, responda las siguientes preguntas marcando con una X la opción de respuesta que más se ajuste a su caso (en ocasiones puede haber más de una respuesta posible).

En todas las preguntas, el periodo de tiempo al que nos referimos es del 17 de marzo del 2020 (fecha en la cual el Instituto es declarado un hospital sólo para COVID-19), al momento en el cual se normalizó la atención de los pacientes con enfermedades reumatológicas. A ese periodo de tiempo lo hemos llamado PANDEMIA COVID-19.

1. **¿Durante la pandemia COVID-19, ha visto afectada de alguna manera, la atención médica Institucional de su enfermedad reumatológica?**
   ☐ No
   ☐ Si

   En caso de haber respondido que Si SE VIO AFECTADA LA ATENCIÓN MÉDICA, señale con una cruz los motivos (puede haber más de uno)

**1.a** Yo decidí no acudir a mi cita programada durante la pandemia	
**1.b** El Instituto canceló mi cita	
**1.c** El instituto reprogramó mi cita para una fecha posterior a la que ya tenía	
**1.d** Requerí de una cita extra y no pude programarla	
**1.e** Requerí de atención por el servicio de urgencias pero no me recibieron	
**1.f** Otros, Señale cual	

2. **Durante la pandemia COVID-19, ¿ha requerido atención médica?**

☐ No

☐ Si, debido a mi enfermedad reumatológica

Especifique la razón: ________________________________

☐ Si, por una razón diferente a mi enfermedad reumatológica

Escriba la razón: ________________________________

3. **Durante la pandemia COVID-19, ¿ha requerido estar hospitalizado?**

☐ No

☐ Si, debido a mi enfermedad reumatológica

Especifique la razón: ________________________________

☐ Si, por una razón diferente a mi enfermedad reumatológica

Escriba la razón: ________________________________

4. **En caso de haber requerido atención médica y/o hospitalización por MOTIVOS RELACIONADOS A SU ENFERMEDAD REUMATOLÓGICA, ¿a quién ha consultado o dónde la recibió?**

**4.a ATENCIÓN MÉDICA**	
**4.a1 ¿A quién consultó?**	
	Médico general
	Reumatólogo
	Médico internista
Otro, especifique:	
**4.a2 ¿En qué lugar recibió la atención médica?**	
	Instituto Nacional de Ciencias Médicas y Nutrición
	Hospital privado
	Otro hospital público
Otro, especifique:	
No aplica/ No necesité atención médica ☐	

**4.b HOSPITALIZACIÓN**

**4.b1 ¿En qué lugar se hospitalizó?**	
	Hospital privado
	Otro hospital público
Otro, especifique:	
No aplica/ No necesité hospitalización ☐	

5. **Durante la pandemia COVID-19, ¿ha tenido la necesidad de comunicarse con algún médico del Departamento de Reumatología? (puede elegir más de una opción)**

☐ No he tenido la necesidad

☐ Si, debido a problemas con mi enfermedad reumatológica

☐ Si, debido a dudas respecto a mi tratamiento reumatológico

☐ Si, debido a dudas sobre la pandemia de COVID-19

☐ Si, debido a otra razón

Escríbalos: ______________________________________

6. **Con respecto a la pregunta anterior, ¿pudo comunicarse al Departamento de Reumatología?**

☐ Si pude

☐ No pude

☐ No tuve necesidad

7. **En caso de haber tenido comunicación con el Departamento de Reumatología, ¿cuál fue el medio que se utilizó? (puede elegir más de una opción)**

☐ No aplica

☐ Correo electrónico del departamento de Reumatología

☐ Redes sociales

☐ Vía telefónica

☐ Otro medio

Escríbalos: ______________________________________

8. **Durante la pandemia COVID-19, usted, personal del Departamento de Reumatología o del Instituto pudieron haber tenido comunicación; nos interesa saber quién hizo el contacto y para qué:**

8.a ¿Pudo usted identificar quién lo llamo?

☐ Si

☐ No

☐ No aplica, no he necesitado comunicarme

☐ Considero que el personal del Instituto no tiene manera de localizarme

8.b ¿Pudo usted identificar para qué se comunicaron con ustedes?

☐ Si

☐ No

☐ No aplica

8.c Seleccione alguna de las siguientes opciones sólo si ha tenido comunicación con el Departamento de Reumatología o el Instituto. (PUEDE ELEGIR MÁS DE UNA OPCIÓN)

	**8.c.1** El Instituto se comunicó conmigo para reagendar mi cita médica y/o de laboratorio
	**8.c.2** El Instituto se comunicó conmigo para darme la consulta médica y/o resultados de laboratorio (**No de la especialidad de reumatología**)
	**8.c.3** El Departamento de Reumatología se comunicó conmigo para reagendar mi cita médica de reumatología y/o el laboratorio correspondiente
	**8.c.4** El Departamento de Reumatología se comunicó conmigo para darme mi consulta de reumatología y/o los resultados de laboratorio
	**8.c.5** Yo me comuniqué con el Instituto para resolver dudas sobre mis consultas médicas y/o mis exámenes de laboratorio
	**8.c.6** Yo me comuniqué con el departamento de reumatología para resolver dudas sobre mis consultas de reumatología y/o de laboratorio
	**8.c.7** Yo me comuniqué con el Instituto para resolver dudas sobre la pandemia de COVID-19
	**8.c.8** Yo me comuniqué con el Departamento de Reumatología para resolver dudas sobre la pandemia de COVID-19

	**8.c.9** Yo me comuniqué con el Instituto para resolver dudas sobre mi enfermedad reumatológica y/o tratamiento reumatológico
	**8.c.10** Yo me comuniqué con el Departamento de Reumatología para resolver dudas sobre mi enfermedad reumatológica y/o tratamiento reumatológico
	**8.c.11** No pude comunicarme con el Instituto
	**8.c.12** No pude comunicarme con el Departamento de Reumatología
	No tuve la necesidad de comunicarme
**8.c.13** Otras razones:	

9. **En caso de haber tenido comunicación con personal del Instituto (no en particular con personal del departamento de Reumatología) , ¿cuál fue el medio que se utilizó? (PUEDE ELEGIR MÁS DE UNA OPCIÓN)**

☐ No aplica

☐ Correo electrónico del instituto

☐ Redes sociales

☐ Vía telefónica

☐ Otro medio

Escríbalos: ______________________________________

10. **En caso de haber tenido comunicación con personal del departamento de reumatología, ¿qué tan útil le resultó?**

**Nada útil**	**Poco útil**	**Moderadamente útil**	**Bastante útil**

☐ No tuve comunicación

11. **Durante la pandemia COVID-19, ¿ha realizado algún cambio en los medicamentos que toma para su enfermedad reumatológica?**

☐ No tengo ningún tratamiento indicado
☐ No he realizado cambios en mis medicamentos
☐ Si he realizado cambios, indicados por un reumatólogo

☐ Si he realizado cambios, indicados por otro médico

☐ Si he realizado cambios, por decisión propia

☐ Si he realizado cambios, por falta de dinero para poder comprarlos

☐ Si he realizado cambios, por problemas para encontrar mis medicamentos (abasto)

Especifique: ¿Cuál o cuáles medicamentos fueron los que no pudo conseguir? ____

☐ Si he realizado cambios, por otra razón

Escríbalos: ____

12. **En caso de haber realizado un cambio en los medicamentos de su enfermedad reumatológica, podría mencionar ¿en qué consistió el cambio? y ¿qué medicamento (s) se modificó (aron)?**
PARA CADA OPCIÓN SELECCIONADA, ANOTE EL NOMBRE DEL MEDICAMENTO A LA DERECHA

☐ No he realizado cambios

☐ Lo suspendí totalmente

☐ Lo suspendí por unos días

☐ Aumenté las dosis

☐ Disminuí las dosis

☐ Tomé otros medicamentos que no tenía indicados

☐ Otros cambios (escríbalos abajo)

____
____

13. **Si tuvo algún problema para conseguir su medicamento, ¿cuál fue la razón?**
PARA CADA OPCIÓN SELECCIONADA, ANOTE EL NOMBRE DEL MEDICAMENTO A LA DERECHA

☐ No aplica

☐ No tenían en las farmacias

☐ No fue entregado por mi seguridad social

☐ No tuve dinero para comprarlo

☐ Otro

Escríbalos: ______________________________________

14. **¿Usted y/o algún familiar con quien usted viva o visite con frecuencia fueron diagnosticados con COVID-19?**

☐ No, ninguno

☐ Si, yo fui diagnosticado

☐ Si, alguno de mis familiares fue diagnosticado

☐ Si, varios fuimos diagnosticados, incluido yo

15. **¿Se les realizó la prueba confirmatoria para COVID-19 a usted o sus familiares con quien usted vive o visita con frecuencia?**

☐ A nadie se le realizó la prueba pues no hubo motivos para ello

☐ No, sólo se realizó el diagnóstico de la enfermedad COVID-19 por las molestias que presentó (aron)

☐ Si, se realizó la prueba para la enfermedad de COVID-19 (toma de muestra por la nariz o por la garganta)

☐ Si, se realizó el estudio de imagen para ver los pulmones (radiografía o tomografía)

16. **¿Cuáles fueron los síntomas que presentó usted (puede elegir más de una opción)**

- ☐ No tuve ningún síntoma o molestia por COVID-19
- ☐ Fiebre mayor a 38° ☐ Dolor muscular ☐ Dificultad para percibir olores
- ☐ Tos seca ☐ Dolor de garganta ☐ Dificultad para respirar
- ☐ Diarrea ☐ Vómito ☐ Cansancio extremo
- ☐ Dolor de pecho ☐ Dolor de cabeza ☐ Cogestión nasal
- ☐ Otras molestias. Escríbalas: ____

17. **¿Durante la pandemia COVID 19, qué tanto siguió las recomendaciones de quedarse en casa el mayor tiempo posible?**

**Siempre**	**Casi siempre**	**A veces**	**Casi nunca**	**Nunca**

18. **En caso de haber contestado a la pregunta anterior (número 17), alguna de las siguientes: "A veces", "Casi nunca" o "Nunca", ¿cuál fue el motivo?**

- ☐ Por mi trabajo
- ☐ Por tener obligaciones familiares
- ☐ No lo consideré necesario
- ☐ No sabía que tenía que guardarme
- ☐ Otros motivos (por favor anótelos abajo)

____

- ☐ No aplica porque me quedé en casa siempre o casi siempre

19. **Durante la pandemia COVID-19, ¿cuáles fueron sus mayores preocupaciones relacionadas a su salud en general o a su enfermedad reumatológica?**

Relacionadas con su **SALUD EN GENERAL**	Relacionadas con su **ENFERMEDAD REUMATOLÓGICA**

☐ No me preocupó mi salud en general ☐ No me preocupó mi enfermedad reumatológica

20. **Durante la pandemia COVID-19, ¿en qué medida el Departamento de Reumatología le ha ayudado a resolver y/o atender esas preocupaciones relacionadas a su salud?**

**Nada de ayuda**	**Algo de ayuda**	**Ayuda moderada**	**Bastante ayuda**	**Mucha ayuda**

21. **Durante la pandemia COVID-19, el Instituto se concentró en atender sólo pacientes con COVID 19 y suspendió temporalmente la atención habitual de todos sus pacientes. ¿Cómo se sintió usted con esa medida?**

____

22. **¿Cómo calificaría la situación de la epidemia COVID-19 que se vive en México?**

☐ Muy grave

☐ Grave

☐ Regular

☐ Poco grave

☐ Nada grave

23. **¿Qué tan probable cree que es que se contagie del coronavirus (COVID-19)?**

- ☐ Altamente probable
- ☐ Muy probable
- ☐ Regular
- ☐ Poco
- ☐ Nada
- ☐ Ya me he contagiado

24. **La epidemia del COVID-19, qué tanto le ha hecho sentir …**

	**Muchísimo**	**Mucho**	**Regular**	**Poco**	**Nada**
**24.1 Ansioso**					
**24.2 Preocupado**					
**24.3 Temeroso**					
**24.4 Alerta**					
**24.5 Deprimido**					
**24.6 Confundido**					
**24.7 Alarmado**					
**24.8 Aislado**					
**24.9 Discriminado**					
**24.10 Aburrido**					

25. **¿La alerta sanitaria afectó la convivencia con su familia?**

- ☐ No
- ☐ Si, positivamente
- ☐ Si, negativamente

26. **¿La alerta sanitaria ha tenido efectos económicos negativos en su familia?**

- ☐ Si
- ☐ No (pase a la pregunta 28)

27. **¿Por qué motivo se ha afectado la economía familiar?**

- ☐ Desempleo de alguno de los miembros
- ☐ Cierre de negocios familiares o no familiares
- ☐ Disminución de trabajo
- ☐ Pérdida de trabajo
- ☐ Falta de pago (por ejemplo, por el cierre de instituciones)
- ☐ Otro. Especifique: ____

28. **Cree que el riesgo de contagio cuando se regrese a la atención habitual en el hospital es:**

- ☐ Muy alto
- ☐ Alto
- ☐ Moderado
- ☐ Bajo
- ☐ Ninguno
- ☐ No sé

MUCHAS GRACIAS POR SU PARTICIPACIÓN

COVID-19 SURVEY (ENGLISH VERSION)

The Immunology and Rheumatology Department of the Instituto Nacional de Ciencias Médicas y de la Nutrición Salvador Zubirán, is interested in knowing some aspects about your general health and your rheumatic disease course during the COVID-19 pandemic. We also want to know how you have faced those health problems during the period when the Institution was a dedicated COVID-19 hospital.

Please complete the following information:

Date:___________________________

Answer the following questions, marking with an X the response option that best suits your case (it might be more than one answer).

For all the questions, the considered period of time is from March 17, 2020 (the Institution was declared a dedicated COVID-19 hospital and the outpatient clinic was closed) to the outpatient clinic (partially) reinstitution. We have called this period of time "COVID-19 pandemic".

1. **During the COVID-19 pandemic, has Institutional health care provision for your rheumatic disease been affected in any way?**
   - ☐ No
   - ☐ Yes

   If you answered YES, then select reasons with a cross (there could be more than one possible reason)

**1.a** I decided not to attend my schedule appointment during the pandemic	
**1.b** The Institution canceled my appointment	
**1.c** The Institution rescheduled my appointment to a later date	
**1.d** I required an extra appointment and could not schedule it	
**1.e** I required an emergency consultation but it was not available	
**1.f** Other reason, please specify	

2. **During the COVID-19 pandemic, have you required health care assistance?**

☐ No

☐ Yes, because of my rheumatic disease

Specify the reason: ________________________________

☐ Yes, for a reason other than my rheumatic disease

Specify the reason: ________________________________

3. **During the COVID-19 pandemic, have you required hospitalization?**

☐ No

☐ Yes, because of my rheumatic disease

Specify the reason: ________________________________

☐ Yes, for a reason other than my rheumatic disease

Specify the reason: ________________________________

4. **If you have required health care assistance and/or hospitalization for REASONS RELATED TO YOUR RHEUMATIC DISEASE, whom have you consulted or where did you receive it?**

**4.a Health care assistance**	
**4.a1 Who did you consulted?**	
	General practitioner
	Rheumatologist
	Internist
Other, specify	
**4.a2 Where did you received the health care assistance?**	
	Instituto Nacional de Ciencias Médicas y Nutrición
	Private hospital
	Other public hospital
Other hospital, please specify:	
Not applicable/ I did not require health care assistance ☐	

4.b HOSPITALIZATION	
**4.b1 Where were you hospitalized?**	
	Private hospital
	Other public hospital
Other hospital, please specify:	
Not applicable/ Did not need hospitalization ☐	

5. **During the COVID-19 pandemic, have you required to contact a physician from the Immunology and Rheumatology Department? (you may choose more than one option)**

   ☐ I have not required it

   ☐ Yes, due to problems with my rheumatic disease

   ☐ Yes, due to doubts regarding my rheumatic disease-related treatment

   ☐ Yes, due to concerns about the COVID-19 pandemic

   ☐ Yes, due to other reasons

   Write them down: ____________________________________

6. **Regarding the previous question, were you able to contact someone from the Immunology and Rheumatology Department?**

   ☐ Yes, I could

   ☐ No, I couldn´t

   ☐ I didn't have the need

7. **In case you had communicated with the Immunology and Rheumatology Department, what was the way used? (you can choose more than one option)**

   ☐ Not applicable

   ☐ Email assigned to the Immunology and Rheumatology Department

   ☐ Social Networks

   ☐ Telephone

   ☐ Other ways

   Write them: ____________________________________

8. During the COVID-19 pandemic, the personnel from the Immunology and Rheumatology Department and/or from the Institution might have communicated with you. We are interested to knowing who made the contact and what was the purpose?

☐ Yes

☐ No

☐ Not applicable

☐ I believe that the Institution staff cannot localize me

8.b Were you able to identify the purpose?

☐ Yes

☐ No

☐ Not applicable

8.c Select one of the following options, only if you have had communication with someone from the Immunology and Rheumatology Department. (YOU CAN CHOOSE MORE THAN ONE OPTION)

	**8.c.1** The Institution contacted me to reschedule my medical and/or laboratory appointment
	**8.c.2** The Institution contacted me to give me a medical consultation and/or laboratory results, not from the rheumatology specialty, (phone-consultation)
	**8.c.3** The Immunology and Rheumatology Department contacted me to reschedule my rheumatology appointment and/or the corresponding laboratory tests
	**8.c.4** The Immunology and Rheumatology Department contacted me to give me my rheumatology appointment and/or laboratory results (phone-consultation)
	**8.c.5** I contacted the Institution for questions about my medical appointments and/or laboratory tests
	**8.c.6** I contacted the Immunology and Rheumatology Department to answer concerns related to my rheumatology appointment and and/or laboratory tests
	**8.c.7** I contacted the Institution to answer questions about the COVID-19 pandemic
	**8.c.8** I contacted the Immunology and Rheumatology Department to answer questions about the COVID-19 pandemic

	**8.c.9** I contacted the Institution to answer doubts about my rheumatic disease and/or rheumatic disease-related treatment
	**8.c.10** I contacted the Immunology and Rheumatology Department to answer doubts about my rheumatic disease and/or rheumatic disease-related treatment
	**8.c.11** I could not communicate with the Institution
	**8.c.12** I could not communicate with the Immunology and Rheumatology Department
	**8.c.13** Other reasons, please specify :
	I did not have the need to communicate with the Institution or with the Immunology and Rheumatology Department

9. In case you have communicated with personnel from the Institution (not in particular with staff from the Immunology and Rheumatology Department), what was the way used? (YOU CAN CHOOSE MORE THAN ONE OPTION)

☐ Not applicable

☐ Email assigned to the Immunology and Rheumatology Department

☐ Social Networks

☐ Telephone

☐ Other ways

Write them: ______________________________________

10. If you have communicated with staff from the Immunology and Rheumatology Department, how helpful was it?

**Not helpful**	**Not very helpful**	**Moderately helpful**	**Quite helpful**

☐ I did not communicate

11. **During the COVID-19 pandemic, have you made any changes to your rheumatic disease-related treatment?**

☐ I do not have any treatment indicated
☐ I have not made any changes to my medications
☐ Yes, I have made changes, indicated by a rheumatologist
☐ Yes, I have made changes, indicated by another doctor
☐ Yes, I have made changes, by my own decision
☐ Yes, I have made changes, because of lack of money to buy them
☐ Yes, I have made changes, because shortage in medication supply

Specify: Which medication(s) were you unable to obtain?
____

☐ Yes, I have made changes, for other reasons

Write reason(s): ____

12. **If you have changed the rheumatic disease-related treatment, could you mention what the change was and the drug affected?**
FOR EACH OPTION SELECTED, WRITE DOWN THE NAME OF THE MEDICATION

☐ I have not made any changes
☐ I totally failed
☐ I suspended for a few days
☐ I increased the doses
☐ I decreased the doses
☐ I took other medications that I didn´t have indicated
☐ Other changes (write them down)

____

____

13. If you had a problem getting your medication, what was the reason?

FOR EACH OPTION SELECTED, WRITE DOWN THE NAME OF THE MEDICATION ON THE RIGHT

- ☐ Not applicable
- ☐ The drug store did not have the medication
- ☐ It was not delivered by my social security
- ☐ I didn't have money to buy it
- ☐ Other

Write them: ______________________________________

14. Have you and/or any family member with who you live or visit frequently, been diagnosed with COVID-19?

- ☐ No, no one
- ☐ Yes, my self was diagnosed
- ☐ Yes, some of my family members were diagnosed
- ☐ Yes, several of us were diagnosed, including my self

15. Have you or any family member with who you live or visit frequently, been tested for COVID-19?

- ☐ No one was tested because there was no reason to do it
- ☐ No, diagnosis of COVID-19 disease was performed only with the symptoms
- ☐ Yes, the test for COVID-19 disease was performed (sampling by the nose or throat)
- ☐ Yes, the imaging study was performed to view the lungs (X-ray or CT scan)

16. What were the symptoms you experienced (you may choose more than one option)

- ☐ I had no symptoms or discomfort from COVID-19 disease
- ☐ Fever greater than 38° ☐ Muscle pain ☐ Difficulty perceiving odors
- ☐ Dry cough ☐ Throat pain ☐ Difficult to breath
- ☐ Diarrhea ☐ Vomiting ☐ Extreme fatigue
- ☐ Chest pain ☐ Headache ☐ Nasal congestion
- ☐ Other, write them: ____

17. During the COVID 19 pandemic, how much did you follow recommendations to stay home as long as possible?

**Always**	**Almost always**	**Sometimes**	**Almost never**	**Never**

18. In case you have answered the previous question (number 17), with one of the following "Sometimes", "Almost never" or "Never", what was the reason?

- ☐ Because of my work
- ☐ Because of having family obligations
- ☐ I did not consider it was necessary
- ☐ I didn’t know I had to stay home
- ☐ Other reasons (please write them down below)

____

- ☐ Not apply because I stayed home “Always” or “Almost Always”

19. During the COVID-19 pandemic, what were your biggest concerns related to your overall health or rheumatic disease?

Related to your **GENERAL HEALTH**	Related to your **RHEUMATIC DISEASE**

☐ I didn't worry about my overall health ☐ I didn't worry about my rheumatic disease

20. During the COVID-19 pandemic, how much help did you get from the Immunology and Rheumatology Department to resolve and/or address these health concerns?

**Not helpful**	**Somewhat helpful**	**More or less helpful**	**Quite helpful**	**Very helpful**

21. During the COVID-19 pandemic, the Institution was a dedicated COVID-19 hospital and temporarily canceled routine care for all outpatients. How did you feel about it?

____

____

22. How would you rate the seriousness of the COVID-19 pandemic in Mexico?

☐ Very high

☐ High

☐ Moderate

- ☐ Low
- ☐ No seriousness

23 How likely do you think you will get the SARS-CoV-2 infection?

- ☐ Very high
- ☐ High
- ☐ Low
- ☐ Very low
- ☐ I have already caught it

24 Regarding COVID-19 pandemic, how much has it made you feel …

	Very much intensity	Much intensity	Middle intensity	Low intensity	Nothing
**24.1 Anxious**					
**24.2 Worried**					
**24.3 Fearful**					
**24.4 Alertness**					
**24.5 Depressed**					
**24.6 Confused**					
**24.7 Alarmed**					
**24.8 Isolated**					
**24.9 Discriminated against**					
**24.10 Bored**					

25 Did the COVID-19 pandemic affect the family-members relationship?

- ☐ No
- ☐ Yes, positively
- ☐ Yes, negatively

26 Did the COVID-19 pandemic had a negative family economic impact?

- ☐ No
- ☐ Yes

27 If you answered yes to the previous question, what were the reasons?

- ☐ Unemployment
- ☐ Closing of family or non-family businesses
- ☐ Loss of work
- ☐ Lack of payment (for example, due to the closure of institutions)
- ☐ Other. Specify: ____

28 How do you rate the risk of getting SARS-CoV-2 infection at outpatient clinic reopening?

- ☐ Very high
- ☐ High
- ☐ Moderate
- ☐ Low
- ☐ I do not know

THANK YOU FOR YOUR PARTICIPATION
