# Supplementary material for: Biopsychosocial factors are associated with impaired sexual function in Mexican patients with rheumatoid arthritis
Source: PLoS One. 2024 Aug 26;19(8):e0305831. doi: 10.1371/journal.pone.0305831 (PMC11346937; doi:10.1371/journal.pone.0305831)
Supplement: Attachment — Submitted filename: Responses to reviewers.docx [file pone.0305831.s003.docx]

**Responses to reviewers**

**Reviewer #1**

I have had the opportunity to review the manuscript entitled “Sexual and reproductive health in Mexican patients with rheumatoid arthritis during the COVID-19 pandemic: A comprehensive approach from the biopsychosocial path “which seems to me to address a relevant, relatively unexplored topic that would be very useful in the comprehensive clinical approach to patients with rheumatoid arthritis, and which seems appropriate to be published in Plos One.

However, although the authors approach the study phenomenon with a pertinent methodological approach, I consider that the manuscript must be improved in a series of aspects of form and substance to be accepted.

**Response.** We appreciate the reviewer's comments.

Below, I expose this series of aspects that I consider should be corrected or refuted with solid scientific arguments by the authors:

First of all, I recommend that the manuscript be reviewed and corrected by a native English speaker, since some paragraphs are difficult to understand due to the writing style used.

**Response.** *The manuscript was already reviewed by an editing service (Grammarly business edition). It has been re-reviewed.*

Title and Abstract:

A first observation is the absence of correlation or inconsistency between the title and the actual content of the study. The title emphasizes the relevance of linking the central phenomena studied with the concurrence of the SARS-Covid 2 pandemic, however, while reading the manuscript I did not find any solid connection to justify these relationships, except the temporal concurrence of the conducting the study with the pandemic, this leads me to the following question:

Did the authors justify that the presence of the pandemic would have an effect on the sexual and reproductive health of patients with RA? In my opinion, I consider that in order to justify the inclusion of this factor in the title, a comparison of the behavior of the dependent variables of sexual function or health should have been carried out before or after the end of the pandemic.

The title also gives weight to the phenomenon of “reproductive health,” however, this variable is barely explored or is explored with unstructured measurement instruments since the instruments used for the core part of the study (sexual function) are specifically focused. to the domain of function (not health, which is broader from the point of view of semantic content) sexual, not reproductive.

**Response.** *We have updated the title of the manuscript to highlight the main results, which are the factors associated with impaired sexual function, and omitted the reference to the COVID-19 pandemic. The second reviewer has a similar request.* *The title has been updated to “Biopsychosocial factors are associated with impaired sexual function in Mexican patients with rheumatoid arthritis.”*

Additionally, from the title and throughout the manuscript the authors use “sexual health” and “sexual function” interchangeably as synonyms, which are not exactly synonymous terms. I consider that for scientific accuracy the authors should standardize the concepts used throughout of the manuscript.

**Response.** We agree with the reviewer that sexual health and sexual function are not synonyms; in fact, sexual function contributes to sexual health. We have been more accurate about the terms used throughout the manuscript. In particular, in the material and methods and results sections, the term "sexual function" is preferred over the term sexual health, consistent with the outcome of interest, that is, impaired sexual function, defined with appropriate tools. However, the reviewer might also have found the term sexual health throughout the text when appropriate and when mentioned by other authors.

Material and methods:

A fundamental problem is the risk of selection bias that the study has in relation to the population included, since the patients studied were defined as having RA “based on the criteria of the treating rheumatologist.” Since its verification does not impose much additional effort to carry out the study, it is currently very difficult to justify a research project in RA based on patients who have not met the EULAR/ACR classification criteria. If it is not possible to modify it, this aspect (potential selection bias) should be included and discussed in the limitations of the study.

**Response.** We disagree with the reviewer. 2010 EULAR/ACR criteria are classification criteria and should not be used for RA diagnosis (Aletaha et al, 2010). As the authors state, “While classification criteria are potentially adopted for use as aids for diagnosis, the focus of this endeavor was not on developing diagnostic criteria or providing a referral tool for primary care physicians…The criteria do not remove the onus on individual physicians, especially in the face of unusual presentations, to reach a diagnostic opinion that might vary from the assignment obtained using the criteria…. Much like other classification criteria, clinicians may be able to diagnose an individual who has not met the classification criteria definition or who has features that are not a component of the classification criteria.” Aletaha et al. also stated in their manuscript that “there is no gold standard for RA diagnosis.” M*ost of the time, in daily practice, rheumatologists establish the diagnosis based on the combination of clinical signs/symptoms, available clinical tests, and knowledge about the epidemiology of the area. (Aggarwal R et al.* *Distinctions Between Diagnostic and Classification Criteria? Arthritis Care Res (Hoboken). 2015 July ; 67(7): 891–897. doi:10.1002/acr.22583). Finally, it is generally accepted that the diagnosis of rheumatic diseases should be individualized because the classification criteria could not include all the phenotypes of diseases, including unusual features or presentations. Diagnosed by board-certified rheumatologists has been used (Young KA et al. Arthritis Rheum 2013).*

An important question to evaluate the appearance of measurement biases: Were the various instruments used, in addition to having been translated into Spanish, transculturated for the Mexican population?

**Response.** FSFI and IIEF have not undergone transcultural adaptation for the Mexican population. We have addressed it as a limitation.

In the paragraph dedicated to the operational definition of the sexual function variable in men: Were the cut-off points defined to categorize the presence of impaired sexual function arbitrary definitions made by the authors for the purposes of the study?; Or have they been previously validated?

**Response.** We used previously published cut-offs.

In this same paragraph, given that the acronym ISF ¿meaning impaired sexual function? This is the first time it is used in the manuscript. It should be expressed non-acronymized, followed by its acronym in parentheses. The same observation applies to the SF acronym in the subtitle of this paragraph.

**Response.** We are sorry for the mistake. We have reviewed the updated version to address the point raised by the reviewer.

Another important point that the authors should reflect on is that the instrument used to evaluate sexual function in men has the specific objective of evaluating the phenomenon of erectile dysfunction, which, although similar, is not exactly the same as sexual function. Depending on sexual preference, a person could have decreased erectile function, but be sexually satisfied with this degree of erectile function. This aspect is also susceptible to being included in the study's limitations.

**Response.** We agree with the reviewer and have addressed the point as a limitation.

An important aspect is that in the Methods section, the variable “(past or present) sexual discomfort” is not operationally defined, although it is used repeatedly in the Results section.

**Response.** We agree with the reviewer, and in the updated version, we provide an operational definition of sexual discomforts and a reference.

Discussion:

It seems too extensive to me, covering aspects of descriptive information of data that are not relevant to the fundamental aspect of the study: the factors associated with altered sexual health in patients with RA, which only occupies 1.5 of the 5.5 pages dedicated to this section of the manuscript.

**Response.** We have updated the discussion section.

Since it was carried out on a population from the same country, the authors should include in the introduction and discussion the paper: Alvarez-Nemegyei J, Cervantes-Díaz MT, Avila-Zapata F, Marín-Ordóñez J. Pregnancy outcomes before and after the onset of rheumatoid arthritis. Rev Med Inst Mex Seguro Soc. 2011 Nov-Dec;49(6):599-604, which addresses reproductive health topics in Mexican women with RA.

**Response.** The suggestion has been adopted.

In relation to the results of the logistic regression analysis carried out, I consider that, instead of being presented as a Forrest-type graph presenting only the variables that reached statistical significance, it should be presented in tabular form with all the variables included in the model, each one with its OR, 95% CI and corresponding p value.

**Response.** The suggestion has been adopted. In addition to the Forrest-type graph (we have conserved as it summarizes the test-based backward selection that was used to define the final model), we have added Table 3, which presents OR and aOR (required by one reviewer) for all the variables included in the model.

**Reviewer #2**

This is a very interesting manuscript that includes a subsection of the population that deserves more attention in research. I congratulate them on their efforts.

there are some topics, though, that I think could improve the manuscript:

**Response.** We appreciate the reviewer's comments.

In general, The authors indicate that the variables that are affected by RA include non SRH factors. However, there are measures that are reported that are more psychosocial factors: The HADS (anxiety and depression), the RAPID-3 (disease activity/severity), the EQ-5D (health related-quality of-Life), the FACIT-F (fatigue) (which I do understand is part of the listed ´biopsychosocial path´, however this can be simplified.

**Response.** We have tried to provide an updated version that is more accurate and highlights relevant results.

Please use ´participants,´ not ´patients´ throughout.

**Response.** We have adopted the suggestion, particularly in the methods and results sections.

Title: should better reflect what is being studied/found in a more simplified way: one example could be Psychosocial factors associated with sexual function of male and female Rheumatoid Arthritis patients of a. xxxx hospital in Mexico City (or something to that effect)

**Response.** We have followed the reviewer's suggestion and updated the title to "Biopsychosocial factors are associated with impaired sexual function in Mexican patients with rheumatoid arthritis."

Abstract:

The writing of the abstract can be improved, especially in the results section to correctly describe results.

**Response.** We have updated the abstract to address the reviewer´s point.

What is impaired SF? The definition is not adequate to understand what you present in the results.

***Response.*** *We have provided a better definition of ISF.*

The dates seem to be missing when the study was undertaken.

**Response.** We have updated the dates.

What is ´multiple logistic regression´? Do you mean Multivariable? You do not present multivariable results, nor do you indicate what is being controlled for. You present bivariable results (at least in the abstract…). Were there no significant results with multivariable? If so this is important to state.

**Response.** We have updated the term multiple to the appropriate term, multivariable. We have updated the section to make the results from the multivariable regression analysis more evident.

In the results section, the results need to be presented more clearly. Example: I do not understand this sentence: Overall, 49.6% of the patients referred to the current family planning method, and 89.7% had children, with a median of 2´.

**Response.** We have updated the whole section.

The measures that are undertaken with males and females (FSFI, satisfaction) should be presented by sex.

**Response.** The suggestion has been adopted.

It wouldn´t be that ´erectile dysfunction was present in 29.4% of males, but instead 29.4% of males reported erectile dysfunction. You´re are not actually measuring the erectile dysfunction but instead the report.

**Response.** The reviewer is right, we have updated the abstract.

´Male` is not a gender, it is sex.

**Response.** We have updated the term.

The OR and p values can be truncated at two numbers after the decimal.

**Response.** The suggestion has been adopted.

% results should be presented with OR values.

**Response.** We are unsure about what the reviewer is asking for, however, we have added OR for the explanatory variables of interest in table 3.

Could there be confounding variables that need to be controlled for (age)?

**Response.** We added age as a confounder variable in Table 3.

Conclusions:

You cannot conclude that RA compromises SRH, you have no control group (non RA). All you can say that some psychosocial factors were found in individuals with RA, and there were differences of participant sex

**Response.** The conclusions have been updated.

Introduction:

You state that WHO recommends using SH (which includes RH), yet you continue to use SRH. Why is this?

**Response.** We agree with the reviewer. We have omitted the acronym SRH and used/assessed as recommended, SH and RH throughout the text.

Authors write: ´RH in RA patients has focused on reduced fertility (30-35) which has been related to the disease process itself [30], therapy [29], gonadal dysfunction [36], physicians’ advice [30, 31], and individual decisions [30, 31]. ´ what do you mean by physicians advice and individual decisions?

**Response.** We have updated the sentence to improve clarity.

This last sentence and the following starting line 86 need to be put together into one. You´ve already said reduced fertility (subfertility should be called ´infertility´ . This one item (infertility) can be included in the previous sentence as ´infertility´ and put the citations together.

**Response.** We have updated the sentence according to the reviewer´s suggestion.

*Methods*

The study was undertaken between 2020 and 2022. Were there still covid instruments being included in 2022?

**Response.** The COVID-19 survey was administered to all the participants.

There is no need to cite STROBE and include it as supplementary material. I believe it is just the journal that needs this when submitting.

**Response.** We are including STROBE as the journal recommends it should be used in observational studies.

The methods include qualitative research. These results are not reflected in the abstract.

How were the quantitative questionnaires administered? Paper format? Electronic?

**Response.** Data related to RH in the abstract was derived from the semi-structured interview. Questionnaires were administered in paper format. It has been updated in the corresponding section.

In the definitions and groups (what is ´groups´?) you only mention impaired sexual function for males and females. This is not a ´reproductive health´ measure per se, but instead by definition, a sexual health measure. Perhaps think about this (as these are you outcome variables) when reworking your title and focus in your abstract.

**Response**. We have renamed the paragraph as “Definitions” and added one more definition (“sexual discomforts”) as requested by the second reviewer. We have updated the abstract, the manuscript, and the title to highlight factors associated with the outcome of interest, ISF in males and females.

Sample size: if you only needed 151 patients, why did you include more than 100 more?

what is the overall population estimated with RA in CDMX?

**Response.** The sample size estimation was based on a hypothesis considering the RAPID-3 effect size odds ratio of 2.5 in a non-normal distribution, using a one-tailed test, 5% significance level, and 80% power. However, we assumed other factors could be associated with ISF, and we might need a bigger sample size to guarantee sufficient power for the association eventually observed. In fact, our hypothesis was not met, and male sex, FACIT-F score, and cohabitation with the couple emerged as significant factors (instead of RAPID-3). In addition, we wanted to have a significant representation of male participants while respecting the male/female ratio observed in Latin America.

*The prevalence of RA in Mexico City is 1%, with 9.210.000 persons censed in the City. (Peláez-Ballestas I, Sanin LH, Moreno-Montoya J, et al. Epidemiology of the rheumatic diseases in Mexico. A study of 5 regions based on the COPCORD methodology [published correction appears in J Rheumatol Suppl. 2011 Mar;38(3):585]. J Rheumatol Suppl. 2011;86:3-8. doi:10.3899/jrheum.100951.*

Results seem to be expressed (at least in the abstract as OR not AOR or aOR which would be adjusted). Furthermore, you do not indicate what you are adjusting for (other than RAPID-3), but surely age must be a confounder when dealing with sexual function (although it is shocking to me- the literature says that starting late 20´s early 30s, women´s sexual function may start to decline!)

**Response.** We have updated Table 3 with OR and adjusted OR. We have added age as a potential confounder.

Results:

NO need to duplicate in written results what is in the table. This section could be rearranged, considering the recommendations below of the tables.

**Response.** The suggestion has been updated.

What is ´middle aged female? This is a vague definition that can mean a lot of things to different people. Median and IQR should be used for age.

**Response.** As recommended, we have omitted the text from the results section. Tables resume descriptive data with median (IQR), including age.

The table 1 needs some better structure. For example, many variables are presented with mean or medians? This needs to be defined better. But there are variables like Religious beliefs (what does this mean? Is it ANY religious beliefs? Is it being spiritual?

**Response.** The table has been updated and explains that data are presented as median (IQR), unless *, which describes the number and percentage of participants.

Religiosity and spirituality are different (Koenig G et al. Review Article. Religion, Spirituality, and Health: The Research and Clinical Implication. doi:10.5402/2012/278730). Patients were directed to answer the question, ¿Do you have religious beliefs, or consider yourself a religious person (not necessarily practicing it)?

Being in a relationship is the same. So it should be set up as categories for some of these variables: Employment (formal is one category and informal is another category), etc.

**Response.** We have updated Table 1 with a better categorization of religiosity and occupation. However, we have adopted a very open relationship approach and avoided fixed categorizations.

Table 2 is perhaps important, but these variables need to be described in light of your outcome variables (and included in the abstract and better woven into the paper). Otherwise it´s just information that is hanging in the air. Are the anxiety and depression scales ONLY covid related? Why are they not part of other tables? Where these

Now I see RH results (these seem to be missing sadly from a lot of the paper)

For the results section- I would organize like this:

**Response.** We are presenting an updated version of the results and tables. The depression and anxiety scales are not necessarily related to COVID-19, while the negative emotions described in the corresponding section are. RH results are described in a specific section and included as potential variables associated with ISF. We hope the updated version will satisfy the reviewer.

Table 1: demographic characteristics with medians(IQR), means(SD) and categories correctly presented

Table two: Outcome variables presented of impaired SF and non-impaired SF(columns) against demographic variables (rows). The covid variables should just be other non-outcome variables but labeled correctly.

Table three: OR with significant variables found in table two.

**Response:** We have incorporated the suggestions in the updated version of the manuscript.

Discussion:

This should be in sync with the more developed results. As it is, it is quite long and wordy, but does not need to be. You need to decide if COVID is going to be main non-outcome variables or not (depending on results), this is not even mentioned in the first paragraph of the discussion.

**Response.** The discussion has been updated and reduced.
