# Supplementary material for: Biopsychosocial factors are associated with impaired sexual function in Mexican patients with rheumatoid arthritis
Source: PLoS One. 2024 Aug 26;19(8):e0305831. doi: 10.1371/journal.pone.0305831 (PMC11346937; doi:10.1371/journal.pone.0305831)
Supplement: Attachment — Submitted filename: RA_r1notes.docx [file pone.0305831.s004.docx]

Thank you for the opportunity to review this manuscript. Although it is much improved from the last version, I still feel there are things that can be improved upon.

Abstract

- In the intro (line 83) you say there are characteristics for the LA region (greater female: male ratio and younger age at presentation, although you show this in the intro (with a citation), this sentence alone in the abstract is a bit distracting. Perhaps an opening statement should be that RA affects sexual function or something more general as you are not focused on comparing LA patients to others in the world but instead the affects on
- The abstract still indicates ´patients´ (the term participants was recommended to use). No need to mention more than the term ´Methods´ in the subsection.
- Idncate in the methods section if questionnaires were provider-administered? Study-team administered? self-administered?
- No need to say 1^st^ and 31^st^ (you can just say September 1 yyyy -January 31 yyyy (same with Methods section in body of document)
- Descriptive statistics and multivariable logistic regression analysis were used. (were used to…? (to study x factors related to y)
- What is FACIT-F.? Please don´t use acronyms without defining.
- The conclusion needs to be more explicit. X, y and z were found to be associated with ISF…. We recommend….or a conclusion of the findings.

Introduction:

- You cite Caucasians and which sex is most affected (please correct to females and males as gender is not as much a factor as sex is)- can you find a citation that looks at the F:M ratio among Latin Americans?

Methods

- Normally the ethics section goes at the end of the Methods section
- It is not clear (line 136) who applied the paper survey. They say administered, how was that administration (this should be discussed in the limitations section as well as any way you apply a survey there are bias’s to how people respond)
- Definitions subtitle could be better titled as ´Measures´
- ´Statistical analysis´ subtitle could be better called ´Statistical Analyses´ as there are more than one.
- I think the terms univariable/multivariable or univariate/multivariate should be homogenized. Right now, there is univariate/multivariable.
- What was the adjustment in the aOR for? (i.e. what did you adjust for)
- Missing data : (line 222) is this only per question? Or if someone said I don´t want to answer to one question then none of their questions were included?
- Line 226´A value of p<0.05 was considered statistically significant.´ seems wrongly placed

Results

- Line 230 missing data needs to be better explained as above (and no need to repeat if this has already been explained in methods)
- Table 1: what are ´self-referring Catholics and self-referring non-religious?´ is this self-reported? If so, could probably drop it as would think all religion is self-reported?
- This table could be better presented as

| Variable |  | N=xxx |  |
| --- | --- | --- | --- |
|  | Category | n | % or median(IQR) |
|  | Category | n |  |
|  | category | n |  |

And some variables could be kept as one (e.g. age)

Please indicate what the * stands for

Table 1 should be presented with male and females as well:

|  |  | All participants | | Male participants | | Female participants | |
| --- | --- | --- | --- | --- | --- | --- | --- |
| Variable |  | N=xxx | % or median(IQR) (should be denoted as median if you present median) | N=xxx | % or median(IQR) (should be denoted as median if you present median) | N=xxx | % or median(IQR) (should be denoted as median if you present median) |
|  | Category | n |  |  |  |  |  |
|  | Category | n |  |  |  |  |  |
|  | category | n |  |  |  |  |  |

Please don´t repeat written results if they are in the table, unless they are some of the main results. The written results can be cut down a lot I think.

Table three could be presented as two extra columns of table 2 (in the same format as my suggestion for Table 1 above), as its best to be able to see OR and aOR against %. Please define what the current table 2 p-values are describing.

Figures 1 and 2: make sure data is not repeated in tables. If it is, please eliminate figures.

Figures 3 (it´s not titled but I think it is figure 3) doesn´tadd much to the data as this is already presented in the table.

Was age added in the model as an adjusting factor? This should be explained in the methods perhaps.

Discussion.

The first paragraph should highlight a few findings from your analyses and the following paragraphs explain these findings further and compare to other research in the same paragraph.

Please check grammar throughout but (line 340, 341 especially)

Your discussion on male participants (lines aprox. 350) is biased because of your low sample size, this should be highlighted.

Fully discuss limitations (some ideas above)
