# Supplementary material for: Biopsychosocial factors are associated with impaired sexual function in Mexican patients with rheumatoid arthritis
Source: PLoS One. 2024 Aug 26;19(8):e0305831. doi: 10.1371/journal.pone.0305831 (PMC11346937; doi:10.1371/journal.pone.0305831)
Supplement: Attachment — Submitted filename: Responses to reviewers.docx [file pone.0305831.s005.docx]

**Responses to reviewers**

**Reviewer #1**

I consider that the authors have carried out all the suggested corrections, or have refuted in a solid argumentative manner those with which they disagreed, so I consider that the manuscript could be accepted.

***Response.*** *We appreciate the reviewer's comments.*

However, before being accepted, there are some specific aspects that require correction or clarification:

The aOR values of the predictor variables that were significant in the logistic regression model that are mentioned in the abstract, in the text of the results and in Figure 3 do not coincide with those shown in Table 3. In fact, none of the aOR and p values in the corresponding column are significant.

***Response.*** *In the previous revision, the reviewer suggested a table (currently table 3) with OR and aOR (95% CI and p values) from all the variables included in the logistic regression model, and not only those that reached statistical significance. We have updated the title of Table 3 to better align with the information provided. In addition, aORs presented in the results section of the abstract are aligned with data from Figure 3. Both summarize only the variables from the multivariable regression model that ended up reaching statistical significance.*

There appears to be an error in placing the value of the decimal point in the confidence interval of the FACIT variable in Figure 3, it is mentioned as (0.92-0.10), when in reality its correct value appears to be (0.92-1.00).

I consider that once these specific situations have been corrected or clarified, the article can be accepted.

***Response******.*** *We are sorry for the mistake. We have updated the Figure 3.*

**Reviewer #2:**

Thank you for the opportunity to review this manuscript. Although it is much improved from the last version, I still feel there are things that can be improved upon.

Abstract

- In the intro (line 83) you say there are characteristics for the LA region (greater female: male ratio and younger age at presentation, although you show this in the intro (with a citation), this sentence alone in the abstract is a bit distracting. Perhaps an opening statement should be that RA affects sexual function or something more general as you are not focused on comparing LA patients to others in the world but instead the affects on.

***Response.*** *We have adopted the suggestion and proposed an updated version.*

- The abstract still indicates ´patients´ (the term participants was recommended to use). No need to mention more than the term ´Methods´ in the subsection.

***Response.*** *We have adopted both suggestions.*

- Indicate in the methods section if questionnaires were provider-administered? Study-team administered? self-administered?

***Response.*** *We have updated the abstract and clarified that questionnaires were self-administered.*

- No need to say 1st and 31st (you can just say September 1 yyyy -January 31 yyyy (same with Methods section in the body of document)

***Response.*** *We have adopted the suggestion.*

- Descriptive statistics and multivariable logistic regression analysis were used. (were used to…? (to study x factors related to y)

***Response.*** *We have updated the sentence to adopt the suggestion.*

- What is FACIT-F.? Please don´t use acronyms without defining it.

***Response.*** *We apologize. We have defined FACIT-F.*

- The conclusion needs to be more explicit. X, y and z were found to be associated with ISF…. We recommend….or a conclusion of the findings.

***Response.*** *We have updated the conclusions following the reviewer´s recommendation.*

Introduction:

- You cite Caucasians and which sex is most affected (please correct to females and males as gender is not as much a factor as sex is)- can you find a citation that looks at the F:M ratio among Latin Americans?

***Response.*** *We have adopted the suggestion. Ref 38 is appropriately cited. Although the paper focuses on treatment, it describes the data from 1093 patients from 14 LATAM countries, of whom 85.3% were females.*

Methods

- Normally the ethics section goes at the end of the Methods section

***Response.*** *The ethics section was moved to the end of the methods section.*

- It is not clear (line 136) who applied the paper survey. They say administered, how was that administration (this should be discussed in the limitations section as well as any way you apply a survey there are bias’s to how people respond)

***Response.*** *All questionnaires were self-administered, and this information was updated as suggested. We have added biases associated with self-reported questionnaires to the limitations section.*

- Definitions subtitle could be better titled as ´Measures´

***Response.*** *We have updated the subtitle.*

- ´Statistical analysis´ subtitle could be better called ´Statistical Analyses´ as there are more than one.

***Response.*** *We have updated the subtitle.*

- I think the terms univariable/multivariable or univariate/multivariate should be homogenized. Right now, there is univariate/multivariable.

***Response.*** *We have adopted the suggestion.*

- What was the adjustment in the aOR for? (i.e. what did you adjust for)

***Response.*** *We have updated the information required in the statistical analyses section.*

- Missing data : (line 222) is this only per question? Or if someone said I don´t want to answer to one question then none of their questions were included?

***Response.*** *We have updated the paragraph.*

- Line 226´A value of p<0.05 was considered statistically significant´ seems wrongly placed

***Response.*** *We have updated the paragraph.*

Results

- Line 230 missing data needs to be better explained as above (and no need to repeat if this has already been explained in methods)

***Response.*** *We have omitted the information from the results section and provided a better explanation in the methods section.*

- Table 1: what are ´self-referring Catholics and self-referring non-religious?´ is this self-reported? If so, could probably drop it as would think all religion is self-reported?

***Response.*** *We agree with the reviewer and have omitted the information.*

- This table could be better presented as

Variable N=xxx

Category n % or median(IQR)

Category n

category n

And some variables could be kept as one (e.g. age)

Please indicate what the * stands for

Table 1 should be presented with male and females as well:

All participants Male participants Female participants

Variable N=xxx % or median(IQR) (should be denoted as median if you present median) N=xxx % or median(IQR) (should be denoted as median if you present median) N=xxx % or median(IQR) (should be denoted as median if you present median)

Category n

Category n

category n

***Response.*** *We have modified Table 1 and incorporated all the suggestions.*

Please don´t repeat written results if they are in the table, unless they are some of the main results. The written results can be cut down a lot I think.

***Response.*** *We have reviewed the whole section, and currently, there are no results duplicated in the text and the tables or figures.*

Table three could be presented as two extra columns of table 2 (in the same format as my suggestion for Table 1 above), as its best to be able to see OR and aOR against %.

***Response.*** *Table 3 responds to reviewer one request to present OR and aOR for ALL the variables included in the multivariable logistic regression model (instead of presenting only those that ended up being statistically significant, which are summarized in Figure 3). For this reason, Table 3 should remain.*

Please define what the current table 2 p-values are describing.

***Response.*** *Table 2 has been updated, and the reviewer's request has been included in the text.*

Figures 1 and 2: make sure data is not repeated in tables. If it is, please eliminate figures.

***Response.*** *In the current version, there are no repeated data.*

Figure 3 (it´s not titled, but I think it is figure 3) doesn t add much to the data as this is already presented in the table.

***Response.*** *We consider Figure 3 should remain as it presents the results (variables that ended up being statistically significant) from the multivariable regression analysis. These are not presented elsewhere.*

Was age added in the model as an adjusting factor? This should be explained in the methods perhaps.

***Response.*** *Yes, it was, and we have clarified it.*

Discussion.

The first paragraph should highlight a few findings from your analyses and the following paragraphs explain these findings further and compare to other research in the same paragraph.

***Response.*** *We have organized the discussion as suggested.*

Please check grammar throughout but (line 340, 341 especially).

***Response.*** *We have used an editing service (Grammarly Business).*

Your discussion on male participants (lines aprox. 350) is biased because of your low sample size, this should be highlighted.

***Response.*** *We agree with the reviewer and have updated the paragraph. We have also assumed that the number of male participants is a limitation of the study.*

Fully discuss limitations (some ideas above)

***Response.*** *We have updated the limitations section.*
