# Supplementary material for: Biopsychosocial factors are associated with impaired sexual function in Mexican patients with rheumatoid arthritis
Source: PLoS One. 2024 Aug 26;19(8):e0305831. doi: 10.1371/journal.pone.0305831 (PMC11346937; doi:10.1371/journal.pone.0305831)
Supplement: Attachment — Submitted filename: Responses to reviewers.docx [file pone.0305831.s006.docx]

**Responses to reviewers**

Could authors explain the differences regarding a previous paper that explored the prevalence of sexual dysfunction in Mexican women with rheumatoid arthritis (Rojo-Contreras et al. Healthcare (Basel) 2022 Sep 21;10(10):1825. doi: 10.3390/healthcare10101825) focusing on the associated factors of sexual dysfunction to establish the main differences regarding this.

***Response.*** *We have included the reference and updated the discussion section to highlight the similarities and differences between both studies.*

In addition to see for the main contributions of the study carried out, author´s should incorporate into the Discussion section the advantages, disadvantages and areas of opportunity for the internal and external validity of the study carried out.

***Response.*** *In the limitations section, we discussed the internal and external validity of the results. In the conclusion, we summarized the study's advantages and areas of opportunity for future research.*
